# Supplementary material for: Coastal flood impacts and lost ecosystem services along Europe’s outermost regions and overseas countries and territories
Source: Nat Commun. 2026 Jan 7;17:188. doi: 10.1038/s41467-025-66391-7 (PMC12780085; doi:10.1038/s41467-025-66391-7)
Supplement: Supplementary file 1 — Description of Additional Supplementary Files [file 41467_2025_66391_MOESM1_ESM.pdf]

Description of Additional Supplementary Files

File Name: Supplementary Data 1

Description: Impacts from coastal floods along Europe's OCTs and ORs under four scenarios ('low-emissions' (SSP1-2.6), 'moderate-emissions' (SSP2-4.5), 'high-emissions' (SSP3-7.0) and 'very-high-emissions (SSP5-8.5)). Variables shown in separate spreadsheets: Expected Annual Damage (damageTOTAL-mEUR), Expected Annual Population Exposed (populationTOTAL-number of people), Expected Annual Flooded Area (floodAreaTOTAL-km^2) and Expected Annual Value of Lost Ecosystem Services (ecoLost-mEUR). The values express the ensemble median projections and the 5th-95th confidence interval (brackets). Data are provided for the years 2020, 2050, 2100 and 2150.
